# A global reanalysis of storm surges and extreme sea levels

**DOI:** 10.1038/ncomms11969

**Published:** 2016-06-27

**Authors:** Sanne Muis, Martin Verlaan, Hessel C. Winsemius, Jeroen C. J. H. Aerts, Philip J. Ward

**Affiliations:** 1 Institute for Environmental Studies (IVM), Vrije Universiteit Amsterdam, 1081 HV Amsterdam, The Netherlands; 2 Deltares, 2600 MH Delft, The Netherlands; 3 Mathematical Physics, Electrical Engineering, Mathematics and Computer Science (EEMCS), TU Delft, 2628 CD Delft, The Netherlands

## Abstract

Extreme sea levels, caused by storm surges and high tides, can have devastating societal impacts. To effectively protect our coasts, global information on coastal flooding is needed. Here we present the first global reanalysis of storm surges and extreme sea levels (GTSR data set) based on hydrodynamic modelling. GTSR covers the entire world's coastline and consists of time series of tides and surges, and estimates of extreme sea levels. Validation shows that there is good agreement between modelled and observed sea levels, and that the performance of GTSR is similar to that of many regional hydrodynamic models. Due to the limited resolution of the meteorological forcing, extremes are slightly underestimated. This particularly affects tropical cyclones, which requires further research. We foresee applications in assessing flood risk and impacts of climate change. As a first application of GTSR, we estimate that 1.3% of the global population is exposed to a 1 in 100-year flood.

## Abstract (web summary)

Protection of coastlines from devastating flooding associated with sea-level extremes is impeded by a lack of continuous records. Here, the authors apply a hydrodynamic modelling approach and present the first reanalysis of tides, surges and extreme sea levels for the entire world's coastline.

## Graphical abstract

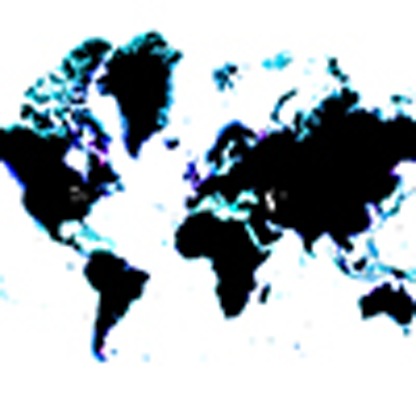

Storm surge, a rise in sea level due to low atmospheric pressure and strong winds, is the main driver of coastal flood events^1^. The most extreme of these events are caused by tropical cyclones^2^, but extra-tropical storms can also produce high sea levels, especially when they coincide with high tide^3^. With over 600 million people living in low-lying coastal areas^4^, coastal floods can have devastating societal impacts. It is estimated that on average 0.8–1.1 million people per year are flooded globally^5^. This is reflected by disasters such as the flooding of The Netherlands and the United Kingdom in 1953, which resulted in over 2,000 fatalities^6^, and led to the construction of a series of flood protection works along the Dutch coast and the Thames Barrier in London. Another more recent catastrophe was the flooding of New Orleans in 2005 due to tropical cyclone Katrina, which resulted in around 1,100 fatalities^7^.

In recent years, coastal flood risk has increased due to population, economic growth^8^ and land subsidence^9^,^10^. To date, socioeconomic development has been the main driver of increasing risk^11^, but in the future sea level rise will be an important driver of increasing risk of coastal floods^5^,^12^,^13^,^14^. To analyse spatial patterns and temporal trends in coastal flood risk, several continental to global scale studies have been carried out^5^,^8^,^12^,^15^,^16^,^17^ based on the extreme sea levels in the Dynamic Interactive Vulnerability Assessment (DIVA) input database^18^,^19^. The extreme sea levels in the DIVA database have been instrumental in many risk assessments, and have provided important insights into which areas face the highest risk, as well as the potential effects of sea level rise and adaptation. However, some applications require time series of sea levels (instead of extreme values), such as: assessing interannual variability; assessing the impact of changes in storm regimes; and the modelling of past events. Such time series can be obtained from tide gauge observations, but many regions at risk have insufficient numbers of tide gauges and/or record lengths available to reliably estimate extreme sea levels. Extreme sea levels vary significantly along the coast due to variability in storminess, coastline shape, and bathymetry^1^. Hence, interpolation between different stations will not accurately capture the spatial variability. Recently, more advanced techniques based on altimetry data have been developed^20^, but the limited length of altimetry records prevent their application to low-probability extreme events^21^,^22^. Due to these data limitations, there is still limited understanding of the global coastal flood hazard, even under current (stationary) climate conditions.

At the regional scale, hydrodynamic modelling is the state-of-art approach to develop consistent and complete sea level reanalyses that can be applied to flood risk assessments^18^,^19^. As the modelling of surges in shallow coastal areas requires a high resolution, generally such a modelling approach has been computationally too costly to apply on the global-scale. The application of unstructured grids (or ‘flexible mesh') in hydrodynamic models make it possible to have a sufficient resolution in shallow coastal areas^23^, while maintaining computational efficiency^24^. These developments in hydrodynamic modelling combined with the increasing availability of global data sets on climate and elevation, make it possible to upscale the hydrodynamic modelling to the global scale.

Here we apply such a hydrodynamic approach for the first time at the global scale and present the Global Tide and Surge Reanalysis (GTSR) data set. To obtain the first near-coast global reanalysis of storm surges (1979–2014), we force the newly developed Global Tide and Surge Model (GTSM)^24^ with wind speed and atmospheric pressure from the ERA-Interim global atmospheric reanalysis^25^. Tides are modelled separately using a recent update of the Finite Element Solution (FES2012) hydrodynamic model (Supplementary Fig. 1 and Supplementary Note 1)^26^. To validate the GTSR data set, we compare the results with observed sea levels from a global data set from University of Hawaii Sea Level Center (UHSLC). Results show that the GTSR time series agree very well with observed sea levels. Extreme sea levels are slightly underestimated, especially those induced by tropical cyclones; this is mainly due to the resolution of the meteorological forcing. To illustrate one of the potential applications of the GTSR data set, we assess global exposure to coastal flooding using a simple inundation model, and find that 1.3% of the global population, equal to 76 million people, is living in the 1 in 100-year floodplain. We foresee various other applications of the GTSR data set, such as: assessing flood risk at the global scale; assessing interannual variability of storminess; providing warning thresholds for operational forecasting models; and assessing the impacts of climate change.

## Results

### Validation of time series

**Figure 1:**
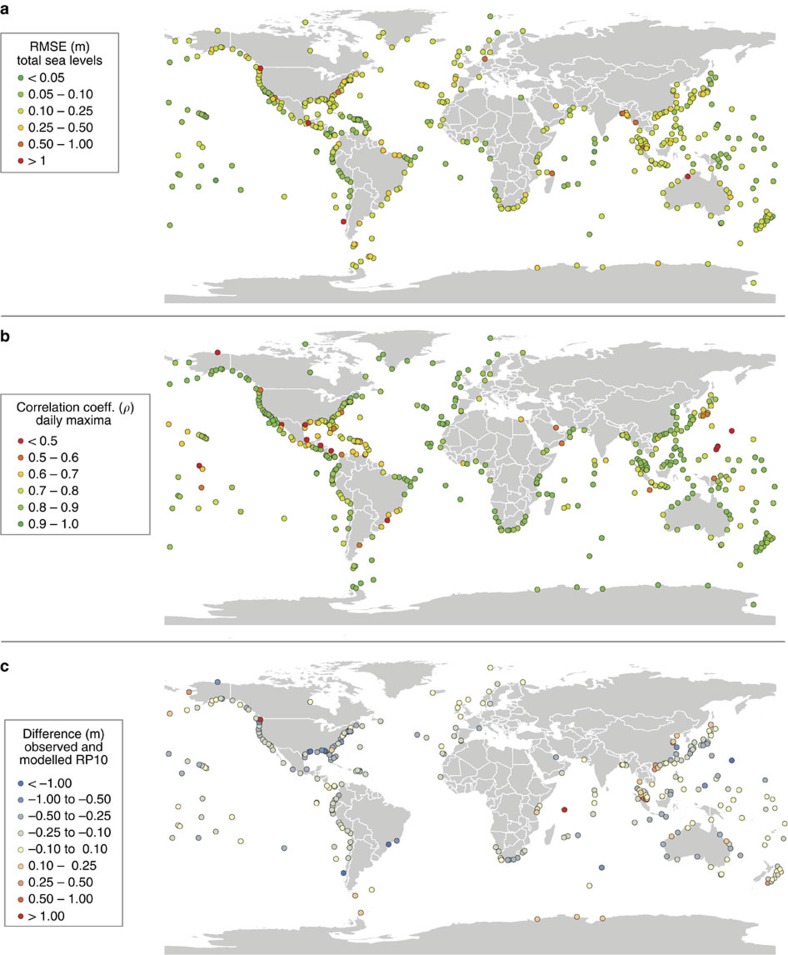
Maps showing the performance of GTSR against observed sea levels. The performance of GTSR shown as (**a**) the RMSE (m) between modelled and observed sea level time series; (**b**) the Pearson correlation coefficient (*ρ*) between modelled and observed daily maximum sea levels; and (**c**) the bias (m) calculated by subtracting the modelled extreme sea levels from the observed extreme sea levels with a return period of 10 years.

**Figure 2:**
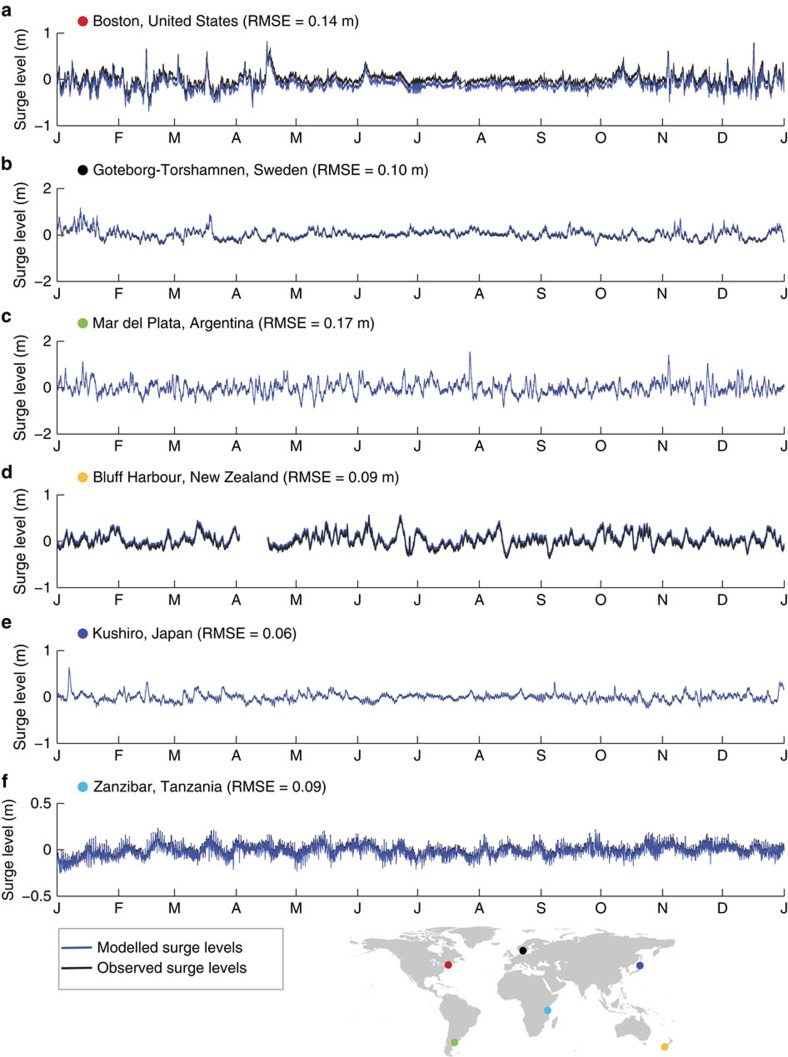
Comparison of the modelled and observed surge levels at selected stations. Comparison of the modelled and observed surge levels for 2007 at six selected stations around the world, (**a**) Boston, United States; (**b**) Goteborg-Torshamn, Sweden; (**c**) Mar de la Plata, Argentina; (**d**) Bluff Harbour, New Zealand; (**e**) Kushiro, Japan; and (**f**) Zanzibar, Tanzania. The coloured dots in the world map indicate the location of the observation stations.

For the total sea level, there is generally a good agreement between modelled and observed sea levels. The root mean square errors (RMSE), based on the time series with a 10 minute temporal resolution, are lower than 0.20m for 80% of the stations (Fig. 1a). The average RMSE across all validation sites is 0.17 m (s.d. is 0.15 m). The performance of the surge levels is even better, with 95% of the stations having a RMSE lower than 0.2 m. The average RMSE is 0.11 m (s.d. is 0.05 m). For illustration, Fig. 2a– shows the modelled and observed surge levels for selected sites around the world. For tidal levels, about 85% of the stations have a RMSE lower than 0.2 m, but the maximum RMSE value is greater than 1 m. The average RMSE is 0.15 m (s.d. is 0.42 m). The large errors (RMSE >1 m) for the tide gauge stations Windham, Victoria and Puerto Montt (red dots in Fig. 1a) are caused by an over- or underestimation of the tidal amplitude. Supplementary Fig. 2a–b shows the RMSEs for the surge levels and tide levels for all observation stations.

**Figure 3:**
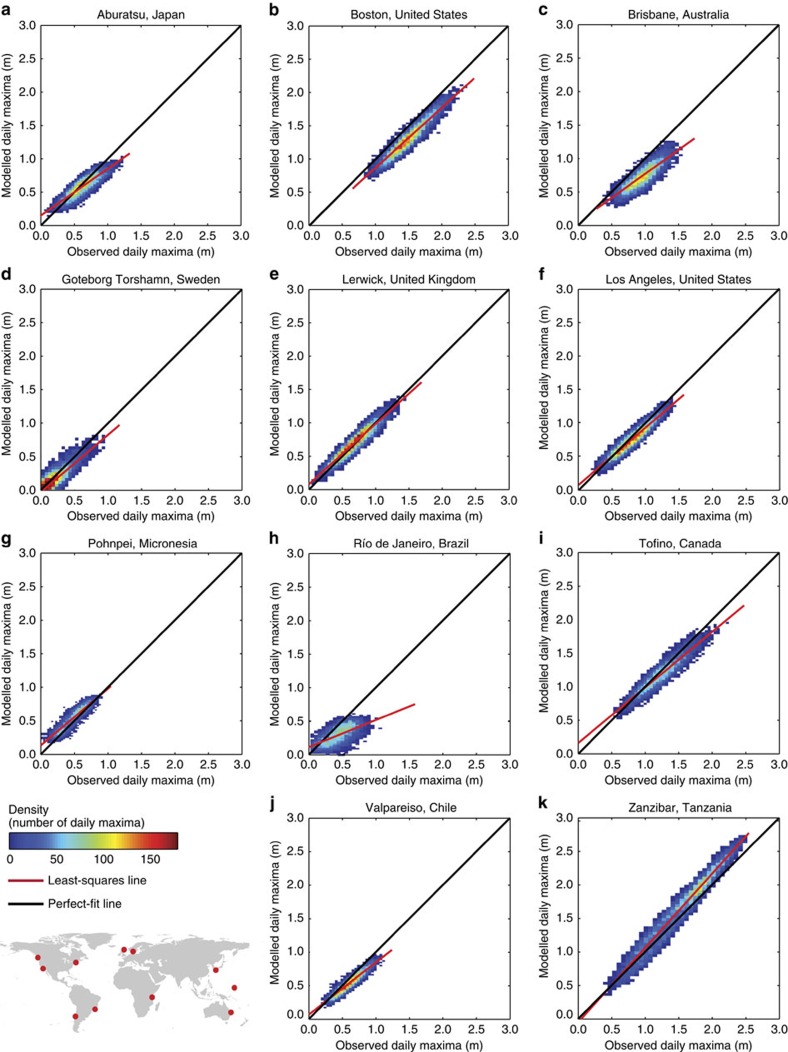
Scatter plots of modelled and observed daily maxima at selected stations. Scatter plots for (**a**) Abaratsu, Japan; (**b**) Boston, United States; (**c**) Brisbane, Australia; (**d**) Goteborg-Torshamn, Sweden; (**e**) Lerwick, United Kingdom; (**f**) Los Angeles, United States; (**g**) Pohnpei, Micronesia; (**h**) Río de Janeiro, Brazil; (**i**) Tofino, Canda; (**j**) Valpareiso, Chile; and (**k**) Zanzibar, Tanzania. Colours indicate the data density for bins with a 5 cm × 5 cm size. The red dots in the world map indicate the location of the observations stations.

To assess whether sea level extremes are also adequately represented in the GTSR time series, we calculate the performance based on daily maxima. Figure 3a–k shows scatter density plots for modelled and observed daily maxima for 12 selected locations. The majority of the daily maxima (orange to red areas in Fig. 3a–k) are close to the perfect-fit line, indicating a good performance. The performance for the majority of these 12 stations decreases for more extreme sea levels (the least-squares line diverges from the best-fit line). The underestimation of extreme sea levels is primarily an issue of resolution (see Discussion section). The average Pearson correlation coefficient is 0.83 (s.d. is 0.14), indicating a good representation of the maxima by the model. Over 75% of the stations have a correlation coefficient higher than 0.75. In regions prone to tropical cyclones (Supplementary Fig. 3a–b and Supplementary Note 2), such as the Caribbean Sea, we obtain correlation coefficients lower than 0.5. The average correlation coefficient in these tropical regions is 0.77, which is significantly lower than the average correlation coefficient of 0.87 in extra-tropical regions (two-tailed Student's *t*-test, *P*<0.05).

### Validation of extremes

**Figure 4:**
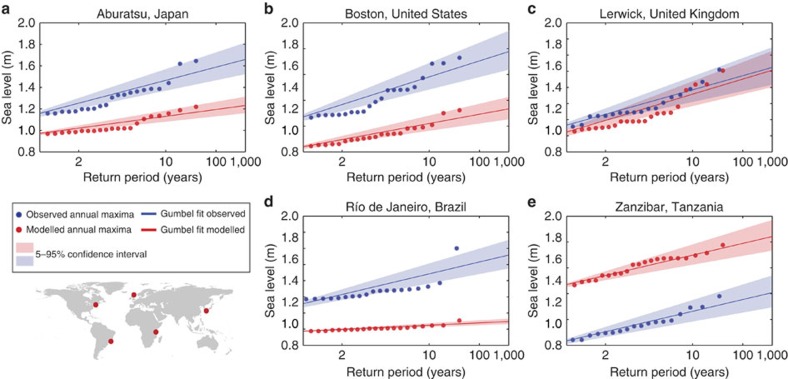
Gumbel plot for the modelled and observed annual maxima at selected stations. Gumbel plots for five selected stations around the world, (**a**) Aburatsu, Japan; (**b**) Boston, United States; (**c**) Lerwick, United Kingdom; (**d**) Río de Janeiro, Brazil; and (**e**) Zanzibar, Tanzania. The annual maxima are plotted against the Gumbel variate, which is equal to x=−ln(−ln(G(y))). The red dots in the world map indicate the location of the observation stations.

To obtain extreme sea levels for various return periods, we fit a Gumbel distribution to the annual maxima using the maximum-likelihood method. The Gumbel plot is shown in Fig. 4a–e for five selected stations. For all stations shown, the annual maxima follow a relatively straight line, indicating a good fit of the Gumbel distribution. Average relative errors for return periods from 5 to 100 years are in the range of 11–14%, increasing with higher return periods. As there is a limited number of stations with observation records longer than the 30 years, we focus on 1 in 10-year extreme sea levels and use all stations that have an observation record longer than 10 years. For 75% of these 144 stations, the absolute error for the 1 in 10-year sea level is lower than 0.3 m (Fig. 1c). On average, the extremes are underestimated by −0.14 m (s.d. is 0.39). Supplementary Fig. 4 shows the results for the 1 in 100-year sea levels. The performance of GTSR is best in areas where extremes are dominated by large, extra-tropical storms, such as Europe, southeast Australia, eastern South-America and northwest North-America. In regions where storm surges are largely induced by tropical cyclones, the mean absolute difference between the extreme sea levels with a return period of 10 years is 0.23 m, which is significantly higher than in extra-tropical regions, where the mean absolute difference is 0.08 m (two-tailed Student's *t*-test, *P*<0.05).

### Application to assess flood exposure

**Figure 5:**
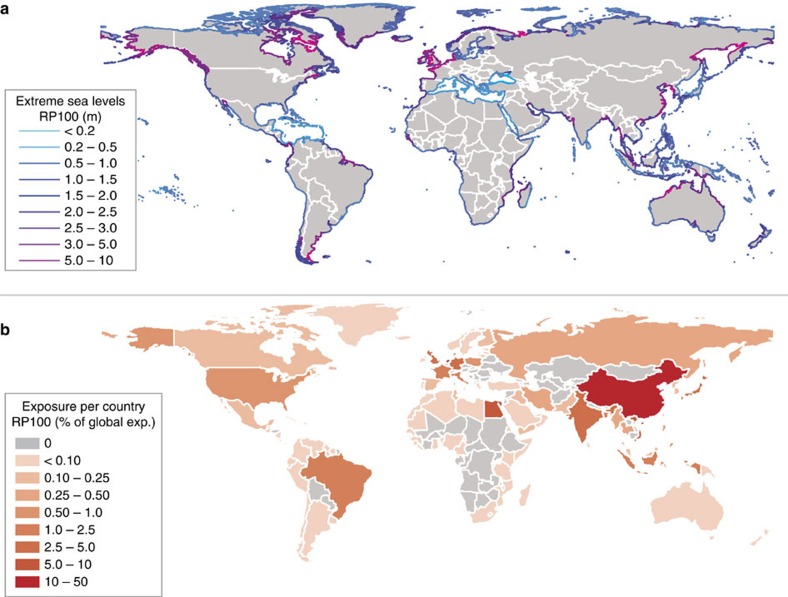
Extreme sea levels with a return period of 100 years and the exposed population. Maps showing (**a**) the height of extreme sea levels with a return period of 100 years (based on the best Gumbel fit) around the entire world's coastline; and (**b**) the estimated exposed population estimates per country (relative to the global exposure) with return period of 100 years.

To illustrate a first application of the GTSR extremes, we calculate the flood hazard (that is, inundation extent) and flood exposure (that is, exposed people) based on 1 in 100-year extreme sea levels. This assessment is based on SRTM elevation^27^ and GRUMP population density for the year 2000 (refs 28, 29). For this first demonstration, we assume no protection from coastal flooding and planar flood levels. Results show that there are large regional variations in the 1 in 100-year extreme sea levels (Fig. 5a)^30^. Relatively high extreme sea levels are found in areas with high tidal amplitude, such as northwest Europe, south Argentina, China and Bangladesh. This spatial pattern agrees with other global data sets^19^,^20^,^31^, and does not change for higher return periods (Supplementary Fig. 5a–b). Combining extreme sea levels with elevation, and assuming no flood protection, shows that major inundation occurs particularly in delta areas in Europe and Asia (Supplementary Fig. 6). The global population exposed to a 1 in 100-year flood is 76 million, which is equal to 1.3% of the total world population (Table 1). The flood exposure map in Fig. 5b shows that China has a particularly large exposure, with 37 million people, which is 3% of the country's population and 53% of the global exposure (Supplementary Fig. 7a shows the absolute exposure). Other countries that have a relatively high exposure include The Netherlands (8.4 million people, 53% of total population), Vietnam (4.7 million people, 6% of global exposure) and Egypt (4.3 million people, 6% of global exposure). Relative to the total national population, The Netherlands and Greenland stand out with 53% and 31% of the population exposed to a 1 in 100-year flood (Supplementary Fig. 7b), respectively; in both these countries the highest concentration of people is found near the coast^28^.

**Figure 6:**
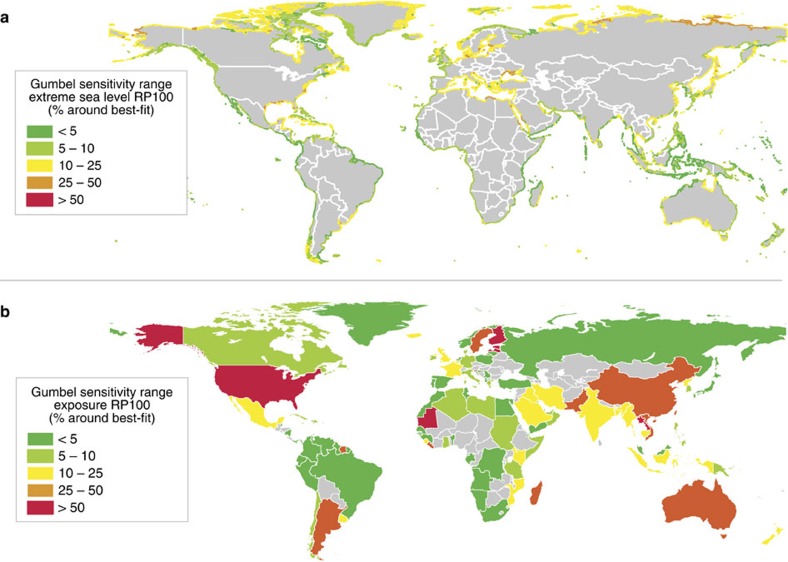
Gumbel sensitivity range for extreme sea levels and flood exposure. Maps showing the uncertainty of the extreme values statistics for the 1 in 100-year return period. The values shown is the range for the 5–95% confidence bounds expressed as a percentage of the value for the best-fit for (**a**) the height of extreme sea around the entire world's coastline; and (**b**) the estimated exposed population estimates per country.

We assess the uncertainty in the extreme value statistics by calculating the 5 and 95% confidence bounds of the Gumbel fit, and express the uncertainty as the percentage difference compared to extreme sea level based on the best-fit. For extreme sea levels with a return period of 100 years, the uncertainty of the Gumbel fit is below 10% for half of the world's coastline (Fig. 6a). The uncertainty is greater than 25% for only 4% of the world's coastline. The largest uncertainty is seen in regions where the extreme values are relatively low, such as the Mediterranean Sea and Caribbean Sea, where a small change in extreme sea level leads to a large increase in relative terms. To assess how sensitive the exposure estimates are to the uncertainty in extreme values statistics, we use the 5 and 95% confidence bounds as input to the inundation and impact model. Globally, the sensitivity to the uncertainty in extreme values is relatively small, with a range of −8 to +21% around the best-fit for exposed population (that is, exposure ranges between 70 and 92 million people). The results per country are shown in Fig. 6, which shows that the results for the USA, Thailand, Mauritania and several countries along the Baltic Sea are particularly sensitive, with uncertainty values greater than 50% of the best-fit. Of the top 10 countries listed in Table 1, the results for China and Vietnam have the largest uncertainty, with exposure estimates ranging from −34% to +39% and −28% to +4% respectively. While the largest uncertainty in extreme sea levels is found along the north coast of Russia, this does not lead to large uncertainty in exposure because the area has a very low population density. On the other hand, an uncertainty in extreme sea levels of 10–50% along the east coast of the USA and <10% along the west coast of the USA leads to uncertainty in the country's exposure estimates between −40% and +49%.

## Discussion

The performance of the GTSR time series is similar to the performance of other hydrodynamic models that cover a large geographic domain. For example, Cid *et al.*^32^ reported a mean RMSE of 0.08–0.10 m for surge levels in the Mediterranean Sea. For a hydrodynamic model covering the entire Australian coastline, Haigh *et al.*^33^ reported mean RMSEs of 0.14 m and 0.05 m for total sea level and surge, respectively. The validation of GTSR shows that extreme sea levels are generally underestimated but that the differences with observed extreme sea levels are rather small (that is, <0.45 m for 90% of the observation stations for the 1 in 10-year sea level). For applications to risk assessment, which make use of data from global digital elevation models (DEMs), this underestimation is reasonable, since the vertical resolution of such DEMs is much greater. For example, the global SRTM DEM has a vertical resolution of 1 m, but its uncertainty is up to several metres^27^. Hence, while acknowledging that the errors can potentially be large in specific locations, we are confident that the GTSR time series (that is, surge, tide and total sea levels) and extremes are a very valuable addition to the global data sets that are currently available. Further benchmarking may be performed by comparing the GTSR extremes with other modelled data sets, such as extreme sea levels in the DIVA database^19^, and regional hydrodynamic models^23^,^34^.

There are several limitations to the GTSR time series and extremes. We aim to update the data set in the future, addressing some of the issues described here. First, the validation shows that extreme values are slightly underestimated. This is an inevitable result of the relatively coarse resolution of the model grid, bathymetry and meteorological forcing (compared with point observations). Extremes in wind speed and atmospheric pressure are smoothed in the ERA-Interim data set due to the temporal (6 h) and spatial (0.75°) resolution. This is particularly problematic for tropical cyclones, which are characterized by strong gradients in atmospheric pressures and wind fields both in time and in space. Our validation results show that the underestimation of extreme sea levels is more severe in tropical areas. However, because of the sparseness and shortness of the available records, the largest tropical cyclone-induced surges are not all included in the available observations^2^. Hence, if we compare the GTSR extremes against reported sea levels induced by tropical cyclones we see larger deviations. For example, the maximum sea levels during tropical cyclone Katrina in New Orleans (2005), and during Typhoon Haiyan (2013), in the Philippines exceeded 8 m (ref. 35) and 4–5 m (refs 36, 37), respectively, while our extreme sea level estimates do not exceed 2–3 m for a return period of 1,000 year. This illustrates that accurately modelling these intense storms requires a much higher resolution than atmospheric reanalysis data can deliver at present day. For updated versions of the GTSR data set, this issue could be resolved by generating localized wind fields based on storm track data and the parametric model of Holland^38^. This is expected to more accurately simulate surge levels resulting from tropical cyclones. However, even when the extreme sea levels are adequately modelled, time series of 36 years contain insufficient number of tropical cyclones to obtain reliable statistics of extreme values. Hence, synthetic resampling techniques are needed to extend the tropical cyclone record to a longer record^33^,^39^,^40^. Including tropical cyclones will lead to higher coastal flood risk, as the most damaging storm surges are often induced by tropical cyclones^41^.

Second, we apply the annual maxima method to obtain the GTSR extremes. Although the method is widely applied, it neglects the fact that sea levels are composed of two independent processes, namely a tide-driven (deterministic) process and a surge-driven (stochastic) process. In addition, data are used inefficiently, as extreme values are estimated based on annual maxima only. To address these limitations, more sophisticated statistical methods, such as the *r* largest or joint probability, could be applied^42^. However, these methods are more sensitive to timing errors and/or temporal or spatial variation. Assuming that tides and storm surges behave independently, the estimates of extreme values could also be made more robust by resampling the surge and tide levels in time to obtain longer time series.

Third, the sea level variations in the GTSR time series are due to gravitational tides and barotropic changes (changes in wind and pressure). Baroclinic effects (density differences) are not considered. While in most parts of the world the generation of extreme sea levels is dominated by tide and surge, in some regions the variations in mean sea level are relatively large^43^ and thus affect the total sea level. For example, this is the case in parts of the Australian coastline^23^. Also, non-linear interactions between storm surges and tides, the effects of waves, and precipitation and river flow are not considered. In this version of GTSR, tides and surges are modelled separately and surge-tide interactions are thus not included, although they are known to be important in shallow water areas with a large tidal range^44^,^45^. Including the surge-tide interaction has improved the model performance for regional hydrodynamic models^46^. For example, in the case of the North Sea, the RMSE was lowered by ca. 40% (ref. 47). However, at this stage this does not apply to GTSM (Supplementary Note 1), as the current version of GTSM is not capable of adequately reproducing tidal characteristics in all coastal regions. Wave setup may increase total sea levels considerably near the coast with the largest contribution in regions with steep slopes^48^. In deltas and estuaries, precipitation and river flow may also contribute to coastal flooding^49^. Including all these processes on a global scale is not feasible at present, but it is important to note that these processes may lead to significantly different extreme sea levels locally.

Also, the impact modelling has some limitations. Aside from limitations of the simple inundation model (see Methods section), the most important limitation is that flood protection is not included in this analysis, and many exposed countries are protected by dikes and storm surge barriers up to a certain design standard. For example, despite its high exposure rates, The Netherlands has very-low flood probabilities as all low-lying areas are protected by coastal defences, some with very high protection standards (1 in 10,000-year return period)^50^. However, Hallegatte *et al.*^12^ estimated protection standards for major cities and found that a city such as Jakarta has a protection standard that equals a 10-year return period. Hence, for many countries listed in Table 1, the protection standard will be lower than a return period of 100 years. Using a similar methodology (planar inundation and no flood protection), but an alternative population database, Jongman *et al.*^8^ estimated that 271 million people are exposed to 1 in 100-year coastal flooding in 2010. On the basis of the similar elevation and population data, but using a somewhat different methodology that includes flood protection, Hinkel *et al.*^5^ estimated that between 160 million people are exposed to a 1 in 100-year flood. The differences in methodology and data make a direct comparison difficult, but our exposure estimate of 70–92 million people seems low compared with previous studies^5^,^8^. In countries prone to tropical cyclones, such as Bangladesh, exposure is underestimated as the tropical storms are responsible for the largest events^2^. Nevertheless, previous studies of global flood risk, including the studies of Jongman *et al.*^4^ and Hinkel *et al.*^7^, are based on the extreme sea levels in the DIVA database^19^. This data set has not been validated at the global scale, but compared to observed sea levels, Muis *et al.*^51^ found that the DIVA 1 in 10-year extreme sea levels in Indonesia are overestimated with on average 0.8 m. Further research could assess differences in flood hazard between DIVA and GTSR and its influence on the estimated flood exposure.

In future research, the GTSR data set could be applied to assess global flood risk, both under current and future climate conditions. Coastal floods may become more severe due to sea level rise and changes in storminess^14^,^52^. While sea level rise may be directly added to the return levels^5^, this is not valid for all regions^53^, and the advantage of the physical based modelling approach is that the sensitivity of this assumption can be tested. Another advantage is that changes in storminess can be assessed using a dynamical approach by forcing the model with future global climate model simulations^54^,^55^. Furthermore, changes in storm duration can be propagated into the full dynamics of storm surge-induced flooding. Such climate change assessments can be used to identify areas that face rapidly increasing risks, which is important for planning disaster risk reduction efforts and to prioritize adaptation efforts^56^. Aside from flood risk applications, the GTSR data set can be used for a variety of other applications. For example, the GTSR time series may be used to: assess changes in storminess^57^; correct for meteorological effects in mean sea level^58^; disentangle the drivers of extreme events in different areas (tidal versus surge dominated)^20^; or assess the influence of interannual variability on risk^59^. As our new time series also includes the duration of flood events, another potential research direction may be the improvement of inundation modelling on a global scale by including flood duration in the inundation modelling. Recent research demonstrated the importance of assessing compound flooding in major US coastal cities^60^. In other delta regions, including compound flood events may also be critical for correctly assessing flood risk^61^,^62^,^63^,^64^. In combination with time series of precipitation or discharge, the GTSR time series could be used to assess compound floods on a global scale. Another application of the GTSM model that is currently being developed is the global operational forecasting system called GLOSSIS^65^, which produces 10-day forecasts of coastal storm surges worldwide. The GTSR extremes could be used to raise warning flags and to identify potential flood hazards. In the future this should not only be based on exceeding a physical threshold, but also include potential impacts of a flood.

## Methods

### General approach

The method to develop the GTSR time series is based on two global hydrodynamic models: GTSM for storm surges, and FES2012 for tides (see Supplementary Fig. 8 for a flowchart). To simulate water levels in all coastal areas, while not generating huge amounts of data, model output is produced for 16,395 locations along the coastline based on the centroids of the DIVA segments database^19^ and the locations of observation stations used for validation. Reduced data sets, useful for extreme value analyses, were generated by post-processing the results into daily maxima as well as annual maxima, for each of the output locations.

### Modelling tides with FES2012

FES2012 is a global tidal model, which assimilates satellite altimetry data. The model is available for download at: http://www.aviso.altimetry.fr. Tidal elevations are distributed on a regular grid of 1/16°. Tides are simulated here with a 10-min time interval. A review on the performance of global tide models by Stammer^66^ shows that FES2012 performs relatively well compared to other global tidal models in coastal areas.

### Modelling storm surge with GTSM

GTSM is based on the Delft3D Flexible Mesh software developed by Deltares^24^. To accurately resolve hydrodynamic equations in topographically complex areas, such as coastal regions, while not decreasing the computational efficiency, it is desirable to locally refine the computational grid^24^. Delft3D FM enables this by allowing the use of unstructured grids. The cell size of the computational grid is dependent on the bathymetry and increases from 1/2° (∼50 km) in deeper parts of the ocean towards 1/20° (∼5 km) in shallow coastal areas (Supplementary Fig. 9). The bathymetric data with a resolution of 1/60° are collected from the General Bathymetric Chart of Oceans^67^ and are interpolated onto the computational grid.

To simulate the sea levels resulting from storm surges, the model was forced with 10 m wind speed and atmospheric pressure obtained from the ERA-Interim data set developed by The European Centre For Medium-Range Weather Forecasts (ECMWF)^25^. The ERA-Interim data set is a global atmospheric reanalysis from 1979 to present day. The meteorological fields are available every 6 h and have a spatial resolution of 0.75 × 0.75°. The meteorological fields are temporally downscaled to 10 min using linear interpolation. The 10-m wind speed is translated into wind stress using a drag coefficient based on Charnock^68^. For consistency with the ECMWF climate model, we applied a Charnock parameter of 0.041. The sensitivity of applying different values for the Charnock parameter is discussed in the Supplementary Note 1.

The model simulations were carried out for each year separately, using a spin-up time of 11 days. Using a parallel setup with 16 cores, each simulation of one year takes approximately 30 h. The different runs were started in parallel, so in theory without other users on the computer cluster, the whole computation can be completed in 30 h.

### Data used for validation

A global data set with observed sea levels is used to validate GTSR. Hourly levels from 472 stations (Supplementary Fig. 10) over the period 1980–2011 are obtained from the archives of the UHSLC (data set is available at http://uhslc.soest.hawaii.edu). All stations are quality-checked by tidal analysis using TideMAT 1.05 (ref. 69). The records of each station are only used when <20% of the data are missing. Each year is analysed separately and mean annual sea level is used as reference date; as such the sea levels are detrended. We subtract the tidal component from the total sea level to obtain the residual level. This component primarily contains the meteorological contribution to sea level (that is, surge level), but may also contain harmonic prediction errors or timing errors. However, we consider these errors as negligible as each station has been inspected visually. Decomposing the total sea level into a tide and surge component enables the separate validation of the two components. For each station with a record longer than 25 years, we also estimate the extreme sea levels for different return periods and compare those with modelled sea levels (see next paragraph).

### Extreme value statistics

To estimate the probabilities of extreme sea levels, we apply extreme value statistics using the annual maxima method^70^,^71^. For each output location, we extract the annual maximum for the calendar years 1979–2014 and fit a Gumbel distribution using the maximum-likelihood method. From the parameterised distribution, we can obtain estimates of sea levels corresponding to selected return periods. We also obtain uncertainty estimates of the parameterised extreme value distribution (that is, 5 and 95% confidence bounds). The Gumbel distribution is often a good approximation of observed extreme sea levels and is frequently applied to estimate return periods^42^. While more advanced statistical methods are available, such as peak-over-threshold or joint probability, the annual maxima method is more robust to temporal and spatial variations, and is thus relatively easy to apply on a global scale.

### Modelling inundation and flood exposure

On the basis of the extreme sea levels, coastal inundation is calculated using a GIS-based planar approach, which uses the extreme sea level and a DEM as input. Inundated areas are defined as areas that have an elevation lower than the water level, and have a direct connection to the sea^5^,^8^. The DEM is obtained during NASA's Shuttle Radar Topography Mission and its original resolution is 1′′ × 1′′ (∼30 × 30 m at the equator)^27^. We use a 30" × 30" (∼1 × 1 km at the equator) average to estimate inundation. Impacts of coastal floods are measured in terms of exposed population using the GRUMPv1 population counts maps for 2000 (refs 28, 29). Flood protection is not included in the analysis and will lead to an overestimation of the flood hazard. Furthermore, the planar approach assumes that a maximum sea level during any event will travel infinitely far land inwards and will therefore overestimate the extent of the floodplain, in particular on coastlines with wide flat areas far land inwards. On the other hand, the spatial averaging of the DEM may result in smoothing of local depressions, causing underestimation of the flood extent in areas with relatively large upward gradients land inwards. Also, coastal cities are often in delta areas where the river may propagate the surge into the hinterland. In this sense, the approach may underestimate the flood extent.

### Data availability

The 1 in 100-year extreme sea levels developed during this study are available in the 3TU.Datacentrum with the identifier ‘10.4121/uuid:aa4a6ad5-e92c-468e-841b-de07f7133786' (ref. 30). Other return periods of the GTSR data set are available on request for scientific purposes.

## Additional information

**How to cite this article**: Muis, S. *et al.* A global reanalysis of storm surges and extreme sea levels. *Nat. Commun.* 7:11969 doi: 10.1038/ncomms11969 (2016).

## Supplementary Material

Supplementary Information Supplementary Figures 1-10, Supplementary Notes 1-2 and Supplementary References.

## Figures and Tables

**Table 1:** Absolute and relative exposed population to a 1 in 100-year flood for the 10 most exposed countries.

**Country**	**Absolute exposure (in millions)**	**Relative exposure (% of population)**
China	36	2.9
The Netherlands	8.4	53
Vietnam	4.7	6.0
Egypt	4.3	6.4
Germany	2.9	3.5
India	2.5	0.3
United Kingdom	2.4	4.1
Japan	2.0	1.6
Bangladesh	1.4	1.1
Belgium	1.1	11
**World**	**76**	**1.3**
