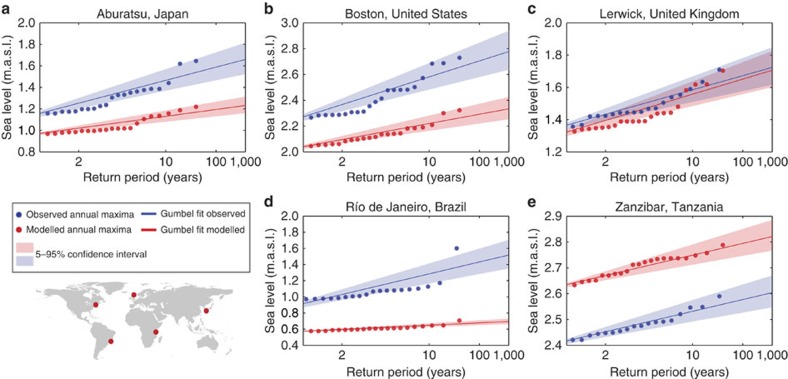# Corrigendum: A global reanalysis of storm surges and extreme sea levels

**DOI:** 10.1038/ncomms12913

**Published:** 2016-09-08

**Authors:** Sanne Muis, Martin Verlaan, Hessel C. Winsemius, Jeroen C. J. H. Aerts, Philip J. Ward

Nature Communications 7: Article number: 1196910.1038/ncomms11969 (2016); Published: 06 27 2016; Updated: 09 08 2016

In Fig. 4 of this Article, the *y* axes in panels ‘b–e' are incorrect. The correct version of Fig. 4 appears below as Fig. 1.

## Figures and Tables

**Figure 1:**