# Supplementary material for: A global reanalysis of storm surges and extreme sea levels
Source: Nat Commun. 2016 Jun 27;7:11969. doi: 10.1038/ncomms11969 (PMC4931224; doi:10.1038/ncomms11969)
Supplement: Supplementary Information — Supplementary Figures 1-10, Supplementary Notes 1-2 and Supplementary References. [file ncomms11969-s1.pdf]

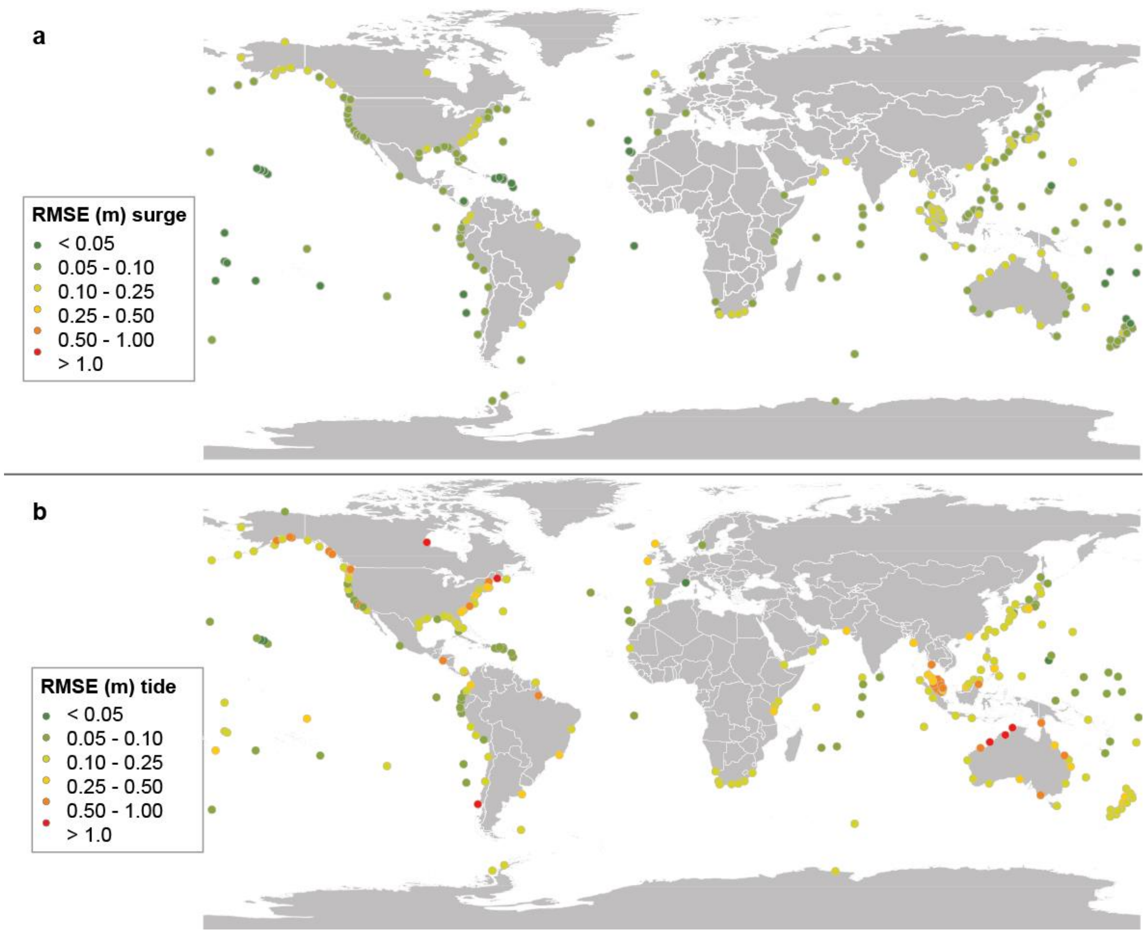

Supplementary Fig. 1: GTSM performance over 2007 for surges and tides. GTSM performance over the year 2007 measured as the RMSE (m) for **a**, surge levels, which are simulated by forcing GSTSM with meteorological fields from ERA-Interim[4]; and **b**, tidal levels where GSTM is forced by the tidal potential[1].

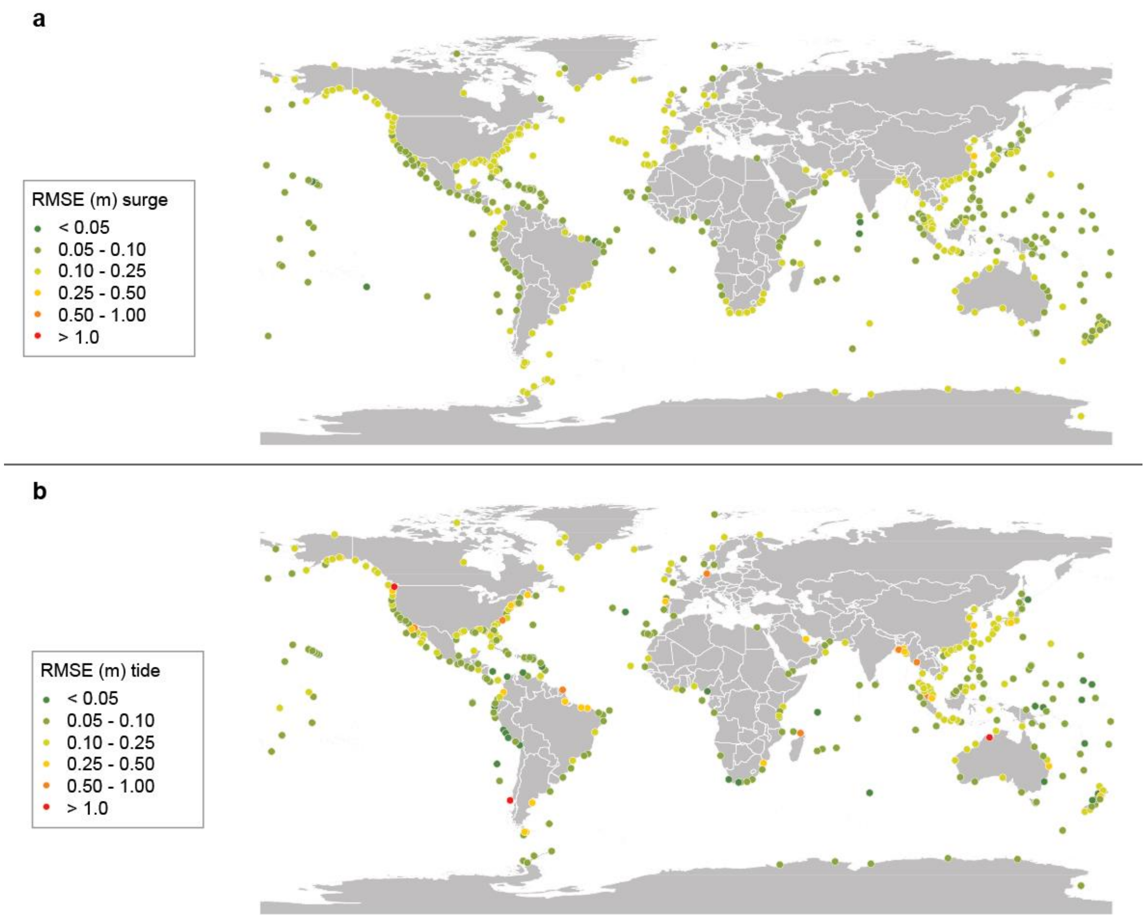

Supplementary Fig. 2: GTSR performance over 1980-2011 as the RMSE (m) for **a**, surge levels, which are simulated by forcing GSTSM with meteorological fields from ERA-Interim[4]; and **b**, tidal levels modelled with FES2012[3].

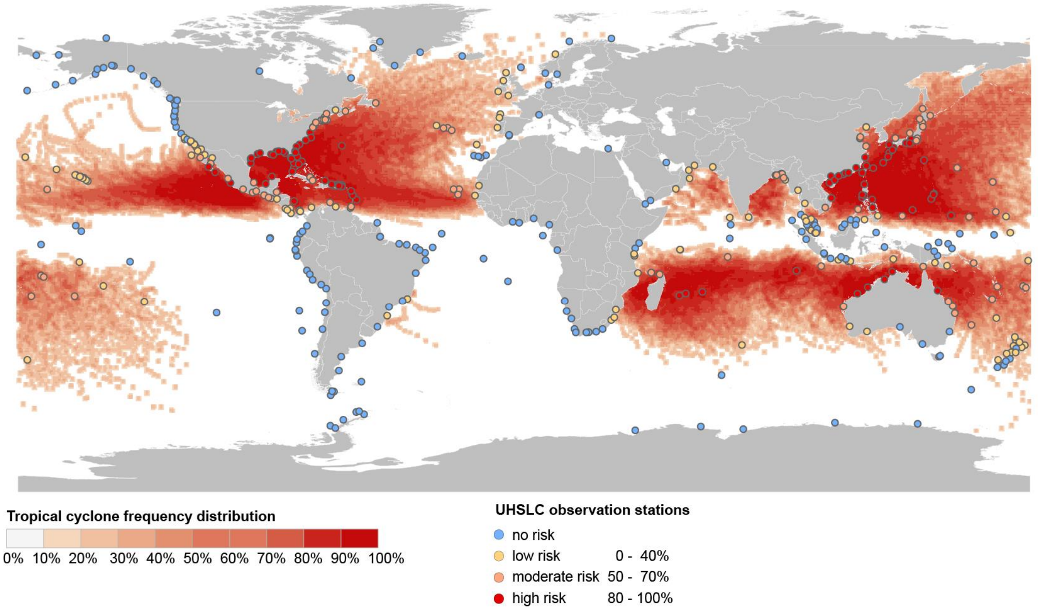

Supplementary Fig. 3: Global map of tropical cyclone hazard frequency distribution, including the frequency distribution divided into four risk categories (no, low, moderate and high risk) for observation stations of UHSLC.

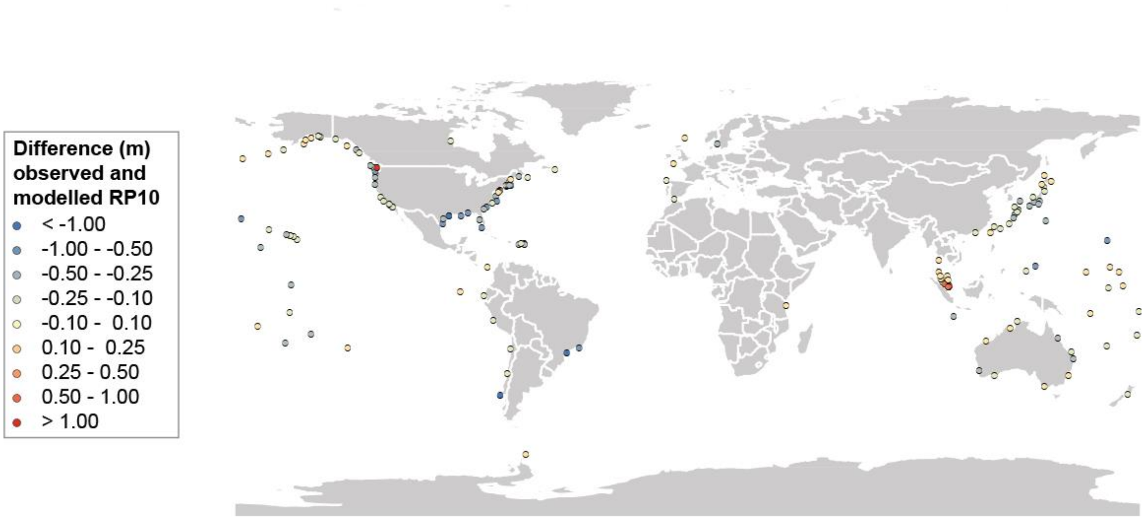

Supplementary Fig. 4: The performance of GTSR showed as the absolute difference (m) between modelled and observed extreme sea levels with return period of 100 years.

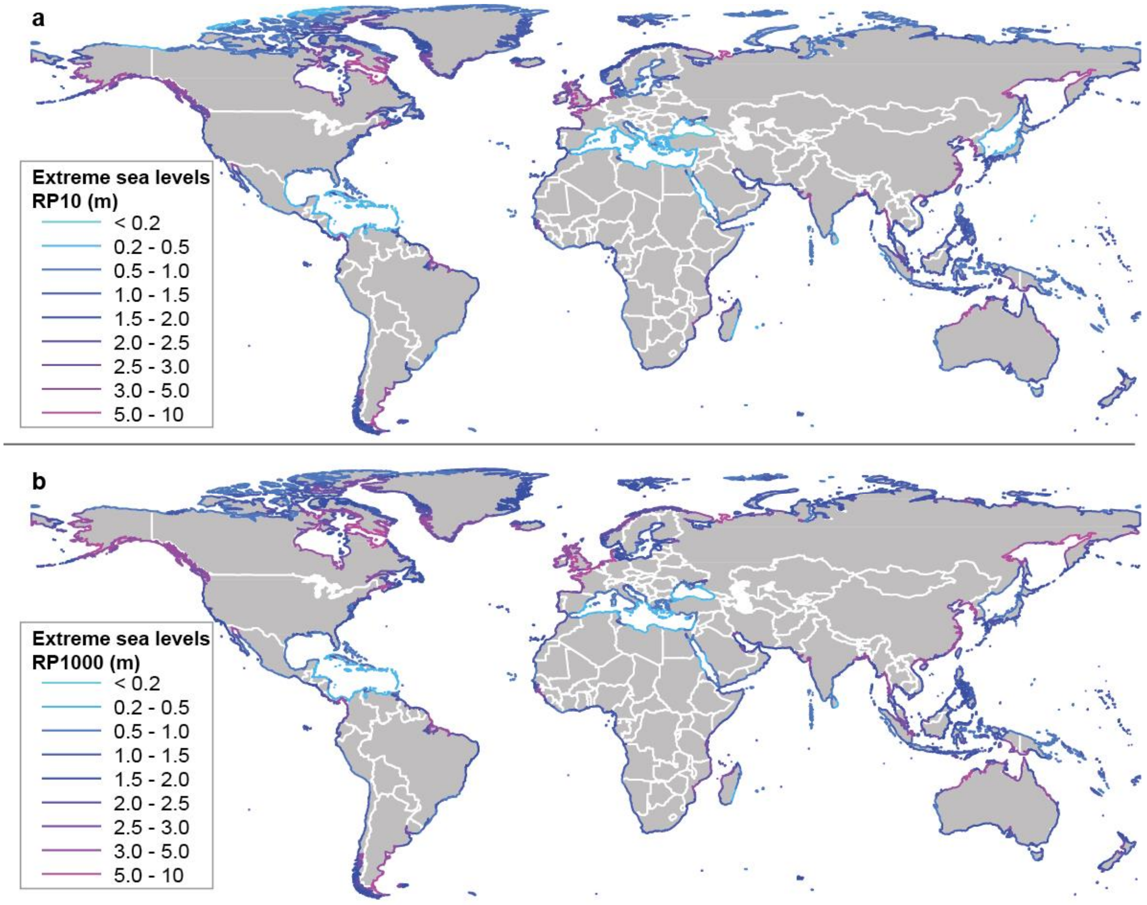

Supplementary Fig. 5: Maps showing the height of extreme sea levels (based on the best Gumbel fit) around the entire world's coastline for **a**, sea levels with a return period of 10 years (RP10); and **b**, levels with a return period of 1000 years (RP1000).

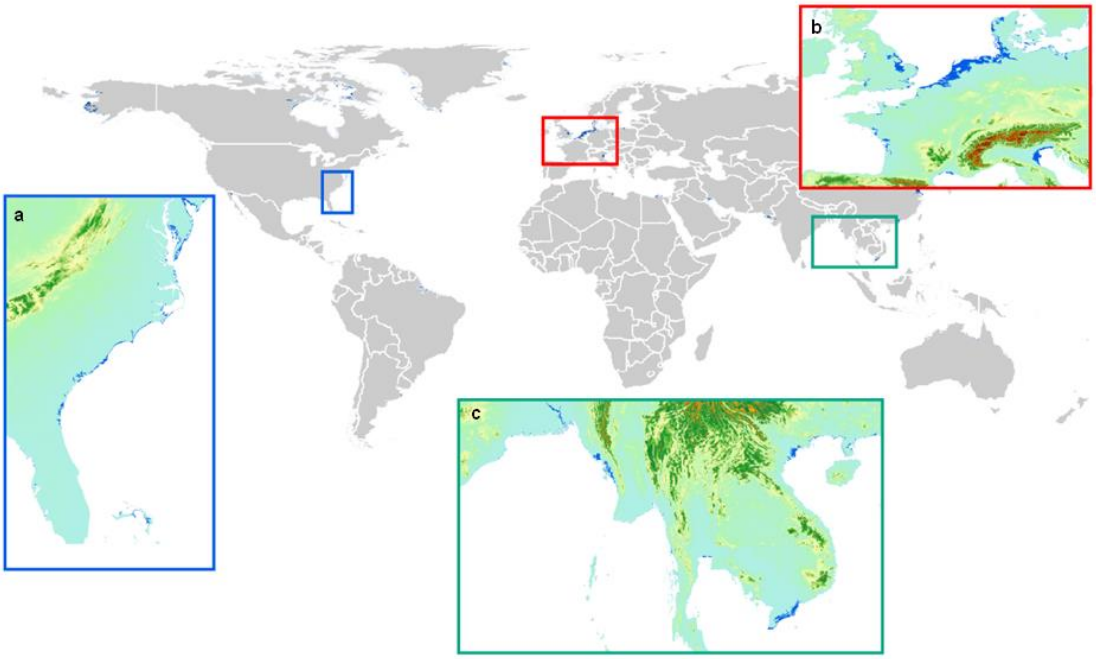

Supplementary Fig. 6: Inundation extent for a flood with a return period of 100 year. The inundation maps shown here are based on a simple bathtub approach without flood protection, and show the inundation extent for a flood with a return period of 100 year for: **a,** the west coast of the USA; **b,** northwest Europe; and **c,** South Asia.

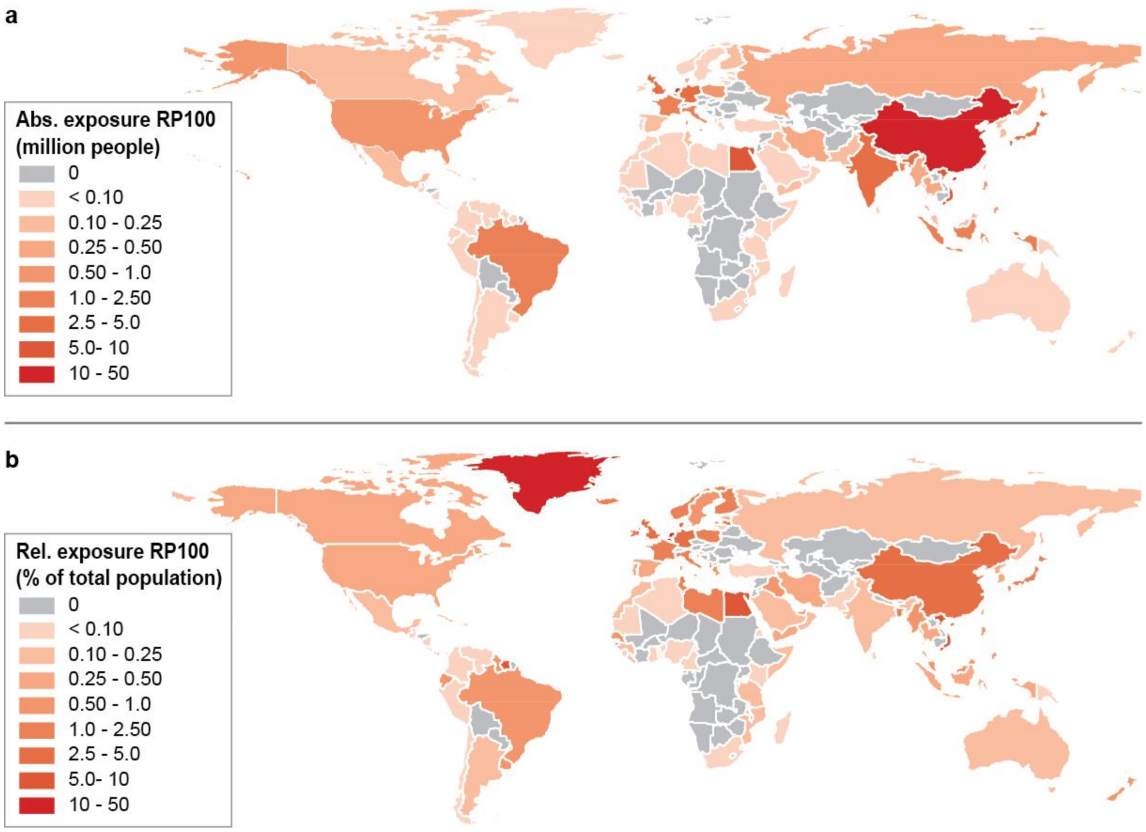

Supplementary Fig. 7: The population exposed to a flood with a return period of 100 year per country expressed as: **a,** the absolute number of exposed people); and **b,** relative to the total population.

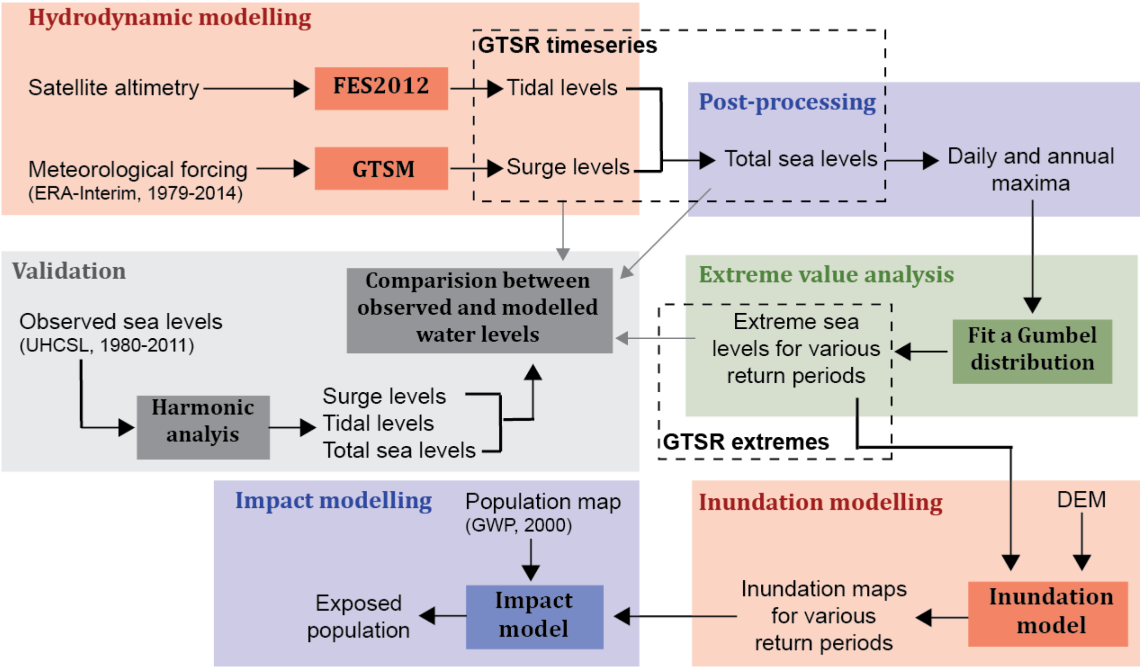

Supplementary Fig. 8: Flowchart of the model approach that was applied to the development and validation of GTSR and the first application for flood risk assessments.

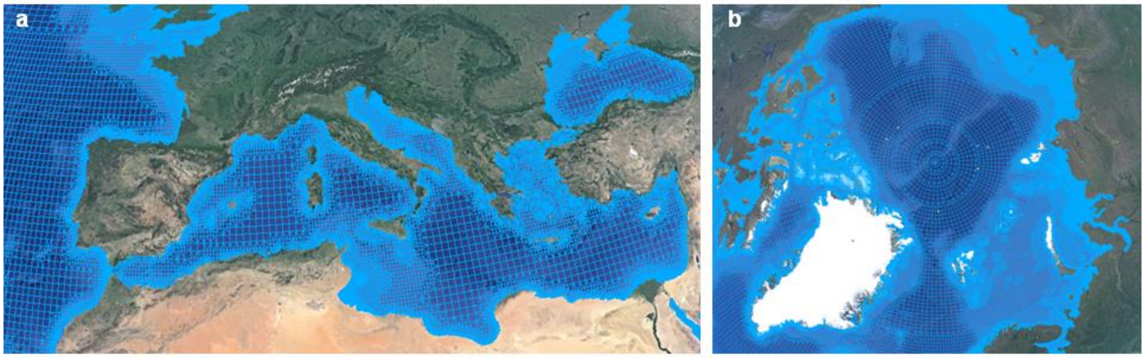

Supplementary Fig. 9: Computational grid of GTSM showing: **a**, the refinement of the grid from the deeper ocean to more shallow areas of the Mediterranean Sea; and **b**, the thinning of the grid at high latitudes.

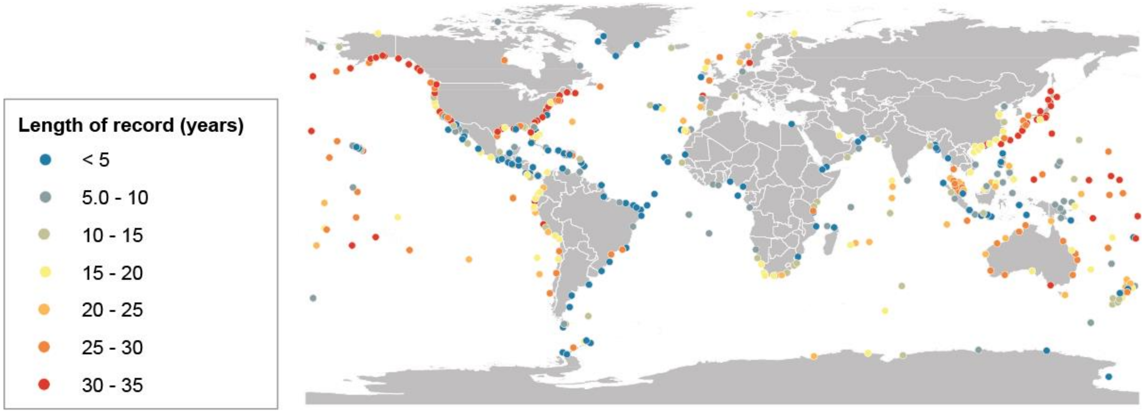

Supplementary Fig. 10: Map showing the locations of the 472 observations stations from the University of Hawaii Sea Level Center (UHSLC) that are used for validation. The colour indicates how many years of observations are available. The UHSLC dataset can be found at http://uhslc.soest.hawaii.edu/.

Supplementary Note 1: First verification of GSTM over 2007

This Supplementary Note provides additional information about the Global Tide and Surge Model (GTSM). GTSM is also capable of simulating tides when forced with the tidal potential[1]. For a first verification of the model, we used 2007 as an example year for which we analyse three model runs, which simulate: the total water level (i.e. tides and surges combined); and the tidal level and surge level separately. The model performance measured as RMSE is 0.32 m (s.d. is 0.28 m), 0.30 m (s.d. is 0.29 m), and 0.10 m (s.d. is 0.04 m) for the total sea level, tide level and surge level, respectively. This shows that the largest model error can be attributed to the representation of tides. For storm surges, on the other hand, there is generally a good agreement between modelled and observed levels. Supplementary Fig. 1 provides more detailed information and shows the performance of GTSM for the different sea level components and for each observation station. The tidal characteristics are not yet adequately reproduced by the model, because not all physical processes that are relevant on a global-scale are included at this stage of the development of GTSM. An important term that is not fully included yet is self-attraction and loading (SAL), which is the gravitational potential of the moving water masses themselves and their capability of modifying the Earth's shape. In the current version of GTSM, we use a simplified approximation of SAL by reduction of the gravitational constant by 10%, implying that the SAL effects are uniform at every place on earth. On a global-scale, SAL has a strong influence on the tidal representation[2], and can lead to changes in the tidal amplitudes of 10% or more and can change phases by 30° or more. Another term that plays a significant role in dissipating tidal energy on a global-scale[2] is the generation of internal tides. Internal tides are produced in stratified waters where tides interact with steep bottom topography (e.g. along the mid-ocean ridges) and stratification in deep water. In the current version of GTSM this is modelled as an additional linear friction component.

Because of weak performance of GTSM to simulate tides at this stage, we decided to rely on FES2012[3] for the tidal component for the full (1979-2014) time series. Using two separate models implies that non-linear interaction between tide and surge, that occur when the surges affect the propagation of the tidal wave and vice versa, cannot be not included. We calculated the surge levels including the surge-tide interaction by subtracting the tide from the total sea level. This showed that the inclusion of surge-tide interaction does not lead to an improvement of the model performance on a global-scale, both model runs for 2007 have a RMSE of 0.10 m (s.d. is 0.04). Overall, the mean RMSE across all stations decreases from 0.30 m (s.d. is 0.29) to 0.15 m (s.d. is 0.16) after implementing the tidal levels from FES2012.

To check whether the Charnock constant has a significant influence on the model performance and for which value the model performs best, we carried out different test runs with a simulation period of one month (January 2007), where we vary the Charnock constant between 0.03 and 0.05. Using a higher parameter value of 0.05 results in a lower RMSE for 92 validation sites. However, generally the improvements are very small (<0.01m). A lower parameter value of 0.03 increases the model performance with more than 0.05 m for 71 stations. Adjusting the Charnock parameter does not lead to an improvement of RMSE on a global-scale, and for consistency with the ECMWF climate model, we chose to maintain the 0.041 value for the Charnock parameter.

Supplementary Note 2: Tropical cyclones

To assess whether the performance of the model declined due to incidence of tropical cyclones, which are only weakly represented in the meteorological fields of the ERA-Interim climate reanalysis, we developed a global map with the tropical cyclone frequency. Such maps have been published in literature, for example by Peduzzi et al.[5], which is also underling the tropical cyclone analysis of the Global Assessment Report on Risk Reduction published by the UNISDR[6]. The data are however not available for download. We therefore used the historical observations of tropical cyclones events (1979 – 2013) from IBTrACS dataset v03r06 [7]. Based on the more than 1,600 storm tracks, we calculated the frequency of the occurrence of tropical cyclones based on a 0.5 degree grid and using the Kernel Density tool, followed by a smoothing filter (Supplementary Fig. 3a). After we created the frequency distribution, we defined four different risk categories: no risk for cells where no tropical cyclones occurred, low risk for the lowest 40% of the frequency distribution, mediate risk for 40-70% of the frequency distribution and high risk for the of the frequency distribution, and assigned these each observation station of the UHSLC dataset (Supplementary Fig. 3b). The frequency distribution is oversimplified as we do not account for different wind speeds and storm size. In general there is however a good resemblance between the maps published in the literature mentioned above and as our only interest is to define whether areas are prone to tropical cyclones or not, we consider the map valid for that purpose. In the validation analysis we compare observations that have no to low risk (the lowest 40% of the frequency distribution) to observation stations that have a moderate to high risk (the highest 50% of the frequency distribution) to tropical cyclones. This means that close to 40% of the observation stations are prone to the occurrence of tropical cyclones.

Supplementary References

1. Accad, Y. & Pekeris, C. L. Solution M2 and S2 Tides in the World Ocenas from a Knowledge of the Tidal Potential Alone. *Phil. Trans. R. Soc. A* **290,** 235–266 (1987).
2. Egbert, G. D. & Ray, R. D. Significant Dissipation of Tidal Energy in the Deep Ocean Inferred from Satellite Altimeter Data. *Nature* **93,** 775–778 (2000).
3. Carrère, L. *et al.* FES 2012: A new global tidal model taking advantage of nearly 20 years of altimetry. in *Proceedings of 20YPRA symposium* 3–8 (2012).
4. Dee, D. P. *et al.* The ERA-Interim reanalysis: configuration and performance of the data assimilation system. *Q. J. R. Meteorol. Soc.* **137,** 553–597 (2011).
5. Peduzzi, P. *et al.* Global trends in tropical cyclone risk. *Nat. Clim. Chang.* **2,** 289–294 (2012).
6. UNISDR. *Globar Assessment Report on Disaster Risk Reduction. From Shared Risk to Shared Value: The Business Case for Disaster Risk Reduction*. (United Nations International Strategy for Disaster Reduction Secretariat, 2013).
7. Knapp, K. R., Kruk, M. C., Levinson, D. H., Diamond, H. J. & Neumann, C. J. The International Best Track Archive for Climate Stewardship (IBTrACS). *Bull. Am. Meteorol. Soc.* **91,** 363–376 (2010).
